# Transcriptional Plasticity of Autophagy-Related Genes Correlates with the Genetic Response to Nitrate Starvation in *Arabidopsis Thaliana*

**DOI:** 10.3390/cells9041021

**Published:** 2020-04-20

**Authors:** Magali Bedu, Anne Marmagne, Céline Masclaux-Daubresse, Fabien Chardon

**Affiliations:** 1 Institut Jean-Pierre Bourgin, INRAE, AgroParisTech, Université Paris-Saclay, 78000 Versailles, France; magali.bedu@bipm.org (M.B.); Anne.Marmagne@inrae.fr (A.M.); Celine.Masclaux-Daubresse@inrae.fr (C.M.-D.); 2 Bureau International des Poids et Mesures (BIPM), Pavillon de Breteuil, F-92312 Sèvres, France

**Keywords:** autophagy, G × E interaction, natural variation, nitrogen stress

## Abstract

In eukaryotes, autophagy, a catabolic mechanism for macromolecule and protein recycling, allows the maintenance of amino acid pools and nutrient remobilization. For a better understanding of the relationship between autophagy and nitrogen metabolism, we studied the transcriptional plasticity of autophagy genes (*ATG*) in nine Arabidopsis accessions grown under normal and nitrate starvation conditions. The status of the N metabolism in accessions was monitored by measuring the relative expression of 11 genes related to N metabolism in rosette leaves. The transcriptional variation of the genes coding for enzymes involved in ammonium assimilation characterize the genetic diversity of the response to nitrate starvation. Starvation enhanced the expression of most of the autophagy genes tested, suggesting a control of autophagy at transcriptomic level by nitrogen. The diversity of the gene responses among natural accessions revealed the genetic variation existing for autophagy independently of the nutritive condition, and the degree of response to nitrate starvation. We showed here that the genetic diversity of the expression of N metabolism genes correlates with that of the *ATG* genes in the two nutritive conditions, suggesting that the basal autophagy activity is part of the integral response of the N metabolism to nitrate availability.

## 1. Introduction

Nitrogen (N), which is incorporated into amino acids, nucleic acids, phytohormones, and chlorophyll, is one of the essential macronutrients for plants. N fertilizers have been widely applied to archive high grain yields in recent decades [1]. However, commercial fertilizers represent the major cost in plant production and less than 50% of soil-applied N fertilizers is used by crops [2]. A major challenge for sustainable agriculture is to improve N use efficiency (NUE) of crop varieties to use less N fertilizer without affecting the yield and quality of harvested products.

Plants adapt their growth and development following the resources of the environment, in particular following the amount of nutrients available in the soil. Frequently, they encounter nitrate deficiency since nitrate has very weak affinity to form surface complexes with soil minerals [3]. Extensive transcriptome studies have characterized the transcriptomic response to N starvation [4,5,6,7,8]. In response to N deficiency, plants modulate root architecture, ammonium and nitrate uptake and assimilation [9,10]. When scarce, ammonium and nitrate uptake mostly proceed through high-affinity uptake systems [11,12,13]. In addition, face with N starvation, plants manage the different intra-cellular nitrogen sources to sustain N economy. The activities of the major enzymes in charge of the nitrate and ammonium assimilation decrease under starvation, while the activities of the enzymes involved in the catabolism of organic nitrogen (glutamate dehydrogenase; GDH) and remobilization (cytosolic glutamine synthetase; GS1) increase to reincorporate liberated ammonium into the whole plant metabolism [4,6,14]. Complex regulations at both transcription and post-translation levels fine-tune these N transporters and N enzymes activities to ensure a tight coordination between N and carbon metabolisms [15].

Extensive natural variations exist for adaptation to environmental changes among individuals belonging to a plant species [16]. Caused by spontaneously arising mutations, these intra-specific phenotypic variations have been maintained over generations by gene flow, genetic drift, and artificial and natural selection. Phenotypic plasticity affects various components of the plant adaptation, such as mineral and nutriment accumulations, primary metabolism, plant growth, and plant development [16]. Using a collection of natural accessions of Arabidopsis (*Arabidopsis thaliana*), we previously characterized the plasticity for growth and N management under normal and nitrogen starvation condition [17]. The vast plasticity existing among accessions defined different types of responses to N stress [17]. Further investigations on the N status of Arabidopsis accessions revealed that the natural diversity affect the management and the storage of various N pools—nitrate, ammonium, amino acids—in the cells [18,19]. The Arabidopsis accessions show different sensibility to nitrate availability and variations in their capacity to produce biomass at the same nitrogen nutrition index. The intra-specific plasticity of the response to low nitrate availability was also observed for N absorption from the media [20,21] and for the management of N fluxes from the rosette to new organs [22]. These studies reveal that the management of the cellular N pools through recycling are key factors for the adaptation of the plants to environmental N availability.

Macrophagy (hereafter referred to as autophagy) is a conserved catabolic process, essential for the removal and recycling of the cytoplasmic components, including damaged proteins and organelles [23]. Autophagy engulfs unwanted cytosolic materials within specialized autophagic vesicles, called autophagosomes, which are subsequently delivered to the vacuole for proteolysis or hydrolysis [24]. Active at very low level, autophagy is highly enhanced in response to many stresses and during leaf senescence [25,26,27,28]. In Arabidopsis, more than 30 autophagy-related (*ATG*) genes have been identified that cooperate in the autophagy machinery for induction, vesicle nucleation, formation of pre-autophagosomal structure, cargo recognition and packaging, vesicle expansion and completion, autophagy-associated protein cycling, vesicle fusion with the vacuole/lysosome, autophagic body breakdown, and recycling of the macromolecules [29]. Growth of the various *atg* mutants under suboptimal conditions indicates that autophagy plays an important role in nutrient recycling, especially during starvation. When the *Arabidopsis* null mutants of *ATG4a/b*, *ATG5*, *ATG7*, or *ATG9*, and *ATG18a* were grown under carbon- or nitrogen-deficient conditions, they exhibited early leaf senescence, accelerated bolting, and reduced seed yield [23,30,31,32,33]. The *ATG18* defective RNAi line and mutants in *ATG5* and *ATG9* were impaired in autophagic flux and showed reduced nitrogen remobilization efficiency [33]. Conversely, the overexpression of several *ATG8* genes increased autophagy activity and enhanced nitrogen remobilization from the rosette to the seeds during the reproductive phase [34].

In response to N starvation, autophagy has been reported to be mainly post-translationally enhanced due to the inactivation of the TOR (Target Of Rapamycin) kinase [35]. However, some reports provide evidence that *ATG4*, *ATG7*, *ATG8*, and *ATG9* are also transcriptionally induced by N and C limiting conditions [30,36,37,38], suggesting a potential and complementary regulation of autophagy at the transcriptional level. The recent studies that screened transcription factors using high-throughput Y1H assays and DNA affinity purification sequencing (DAP-seq) revealed several regulator candidates for *ATG8* genes [38,39]. Among these candidates, the TGA9 basic leucine-zipper protein (bZIP) transcription factor was shown to up-regulate the expression of several *ATG8* genes by binding to their promoters [38]. TGA9 enhanced the expression of *ATG8b* and *ATG8e* in response to sucrose starvation and to osmotic stress. However, the role of ATG9 in the control of autophagy under N starvation was not reported. In the list of transcriptional factors binding the promoters of *ATG8a*, *ATG8b*, *ATG8e* and *ATG8f*, only *TAG1* and *TAG4* are known to be regulatory components of the nitrate response in roots [40]. Furthers investigations are needed to identify the transcriptional regulators of autophagy in the rosettes of plants submitted to N starvation.

The aim of our study was to assess the transcriptomic plasticity of *ATG* genes in Arabidopsis genotypes responding differentially to N starvation. We measured the transcriptional levels of two sets of genes that correspond to *ATG* genes and N metabolism-related genes, in nine natural accessions of *Arabidopsis thaliana* grown in hydroponic cultures under non-limiting supply or submitted for 24 h to nitrate starvation. By using this approach, we compared the accessions following their transcriptomic responses to N stress. The characterized transcriptional plasticity of *ATG* genes under stress and non-stress conditions suggested the existence of natural diversity of basal autophagy. We showed that the global expression of *ATG* genes correlated with expression of genes involved in the assimilation of ammonium.

## 2. Materials and Methods

### 2.1. Plant Material

*Arabidopsis thaliana* accessions used in this study were obtained from the Versailles stock center (http://publiclines.versailles.inra.fr/). The nine genotypes (*Akita*, *Bay-0*, *Bur-0*, *Can-0*, *Col-0*, *Cvi-0*, *Edi-0*, *Sakata*, *Tsu-0*) are part of the core collection of 24 accessions selected by McKhann et al. [41] on the basis of genetic variability.

### 2.2. Plant Growth Condition

Seeds were surface sterilized by using ethanol–‘bayrochlor’ (95/5%, *v*/*v*) prior to stratification in water at 4 °C for 3  days. Each seed was sown using a toothpick on the top of one cut Eppendorf tube filled with 0.7% Daishin agar (Duchefa Biochimine, Haarlem; The Netherlands). Tubes were inserted into 96-wells trays filled with demineralized water. After 3 days in the darkness at 4 °C, the trays containing tubes were transferred to a growth chamber in short days with 21 °C day and 17 °C night temperatures. Relative humidity in the growth chamber was 65%. The photon flux density was 165 μmol m^−2^ s^−1^. On the seventh day of growth, seedlings were transferred to plastic tanks, each with the capacity to grow 104 plants. Each genotype was represented by eight plants per nutrition condition. These plastic tanks were filled with 33.0 L of nutrient solution. The plants were cultivated hydroponically for 35 d (the entire vegetative growth). A short photoperiod (8 h day) was chosen to prevent early flowering.

One set of plants was fed on complete nutrient solution containing 4 mM nitrate as the N source (2 mM KNO_3_, 1 mM Ca(NO_3_)_2_, 0.15 mM KH_2_PO_4_, 0.15 mM MgSO_4_, 0.3 mM CaCl_2_) for 35 days. In the second condition, a set of control plants that were maintained on the complete nutrient solution for 34 days were fed for 24 h a complete N starvation with 0 mM N0_3^−^_ solution (0.15 mM KH_2_PO_4_, 0.5 mM K_2_SO_4_, 0.15 mM MgSO_4_, 0.18 mM CaCl_2_). All nutrient solutions contained microelements (15 μM MnSO_4_, 10 μM H_3_BO_3_, 3 μM ZnSO_4_, 0.01 μM CuSO_4_, 0.1 μM Na_2_MoO_4_, 22.3 μM Na-EDTA, 22.4 μM FeSO_4_, 0.01 μM CoCl_2_, and 0.5 μM KI). The pHs of the solutions were around 5.6. Solutions were renewed once during each week of culture up to harvest.

Two successive cultures were carried out to produce independent replicates of the experiment.

### 2.3. RNA Extraction and Gene Expression Analysis

Rosette leaves from each biological replicate were ground separately to a fine powder in liquid nitrogen and stored at −80 °C until use. Approximately 200 mg of the leaf powder were used for each extraction and added to 2 mL of TRIZOL^®^ reagent (Invitrogen, Thermo Fischer Scientific, Les Ulis, France). Extractions followed manufacturer’s instructions, resuspending the final pellet in 100 µL of sterile water. RNA concentration was checked on a Nanodrop instrument and integrity was checked on a 1% agarose gel. Genomic DNA was removed using RQ1 DNAase (Promega France, Charbonnières-les-Bains, France) according to the manufacturer’s instructions, and removal was verified by PCR using Niia primers (Table S1). cDNA was synthetized from 2 µg of RNA using olido dT and M-MLV RNase H-Reverse Transcriptase (Promega) according to the manufacturer’s instructions. RNA and cDNA was stored at −80 °C until used.

Real time PCR was performed in a Roche Lightcycler using qPCRBIO Syber Green (Thermo Fischer Scientific, Les Ulis, France). Reactions were in 20 µL containing 20 ng of cDNA, 10 µL of Sybergreen, 2.4 µL of sterile water and 0.8 µL of each forward and reverse primer. Reactions were performed with a cycling program of: 95 °C for 120 s, followed by 40 cycles of 95 °C, and 72 °C for 30 s followed by a melting curve form 55 to 98 °C to verify primer specificity. All the primers used are listed in Table S1. Data were analyzed using the 2^−ΔΔct^ method [42]. For all RT-qPCR analyses, *APT1* (*At1g27450*), *ACT2* (*At3g18780*) and *PP2AA3* (*At1g13320*) were used as reference genes (Table S1). The quadratic mean was used to define a synthetic reference gene, which took into account the variation in expression of the three reference genes.

### 2.4. Statistical Analysis

Three-way ANOVA (R software package) was used to assess the effects of the experiment, genotype, nutritive condition, and the genotype by nutrition (G × N) interaction on the trait variation. Each contrast between the control condition and the other conditions was tested by using estimated marginal means with the function contrast of the R emmeans package. Pairwise differences between genotypes were tested by using estimated marginal means with the function CLD of the R emmeans package.

Data were also treated by hierarchical clustering using the R package pheatmap and by Principal Component Analysis (PCA) using the R package FactoMineR.

## 3. Results

### 3.1. Variatons in GLN1.1, Nii, GDH, GLU1, and GLT Gene Expressions Are Good Markers to Characterize the Genotype-Response to N Starvation

**Figure 1:**
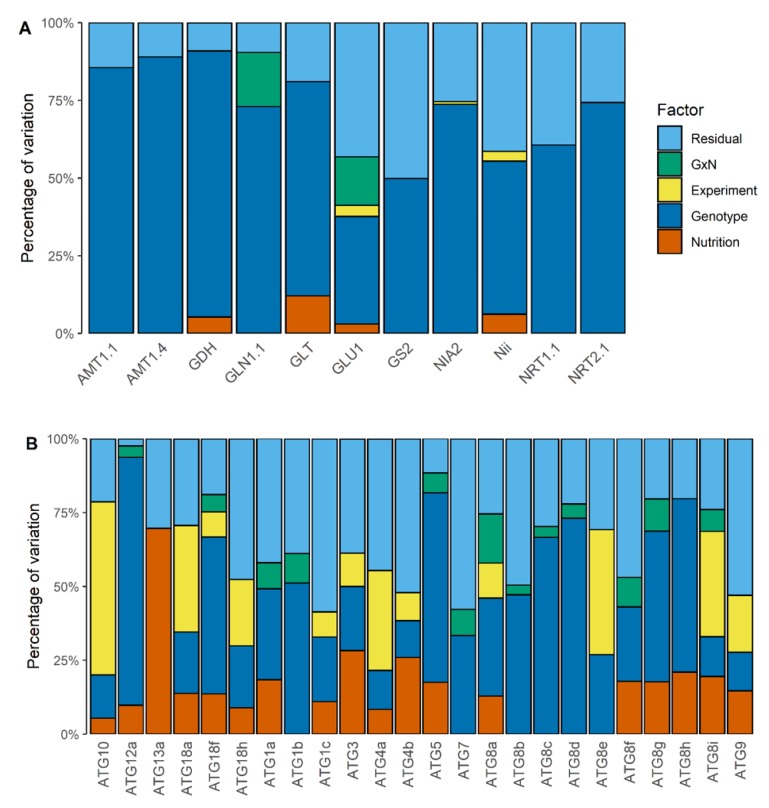
ANOVA of transcript abundance variation for N metabolism-(**A**) and autophagy-(**B**) related genes among the Arabidopsis accessions. Models are adjusted within are tested an experiment repeat effect (Experiment), a nutrition effect (Nutrition), a genotype effect (Genotype), a genotype by nutrition interaction factor (G × N) and residual error (Residual). Only significant effects are showed (*p* < 0.05).

To determine the degree of the response to nitrate starvation for each accession, we measured the relative expression of 11 genes related to N metabolism in their rosette leaves (Supplementary Table S3). The gene set included genes encoding for nitrate and ammonium transporters (*NRT2.1*, *NRT2.1*, *AMT1.1* and *AMT1.4*), enzymes of nitrate reduction (*NIA2*, *Nii*) and enzymes of ammonium assimilation and recycling (*GS2*, *GLN1.1*, *GDH*, *GLT*, and *GLU1*). Thee-way ANOVAs were carried out to test the main factors on the expression variations, such as accession (i.e., genotype), nutrition and experiment, and their potential interactions, such as the genotype by nutrition (G × N) interaction. ANOVA results revealed that the genetic factor was the highest source of variation for all the studied genes (Figure 1A). It explained 35% to 89% of the observed variation.

**Figure 2:**
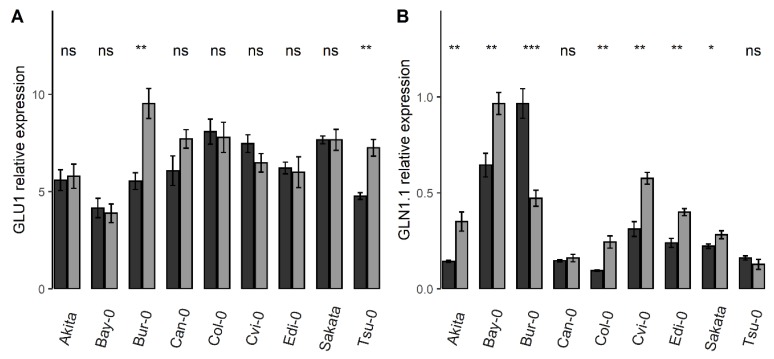
The relative expression of N metabolism-related genes varied depending on accessions and N conditions. Relative expression of *GLU1* (**A**) and *GLN1*.*1* (**B**) illustrating the differential responses to starvation among accessions (means ± SE). Stars indicate the significance of the difference between the means of the control and of the N starvation condition for each accession: ns non-significant, * *p* < 0.05, ** *p* < 0.01, *** *p* < 0.001, *n* = 8. Dark and light greys show the plant in the control and N starvation conditions, respectively.

Besides genetic effects, variations of expression levels for genes linked to nitrate availability were also identified. For four genes, the relative expression was significantly different between the control and starvation conditions (Table 1). The relative steady state transcript levels of *Nii*, *GDH1*, *GLU1*, and GLT were higher (from 12% to 31%) under the N starvation than in the control condition. The enzymes encoded by these four genes are well known to be involved in ammonium assimilation, and these genes are known to be sensitive to the cellular ammonium concentrations. Only *GLU1* and *GLN1.1* showed a significant G × N interaction in ANOVA, suggesting that the accessions responded differently to nitrate starvation with at least these two genes. The relative expression of *GLU1* was not modified by nitrate starvation, except for the accessions *Bur-0* and *Tsu-0* in which N starvation enhanced the relative expression of *GLU1* (Figure 2A). The relative expression of *GLN1.1* was higher in the starvation condition compared to the control condition in most of the accessions except for *Bur-0*, and remained extremely low irrespective of N conditions in *Can-0* and *Tsu-0* (Figure 2B).

We deduced that the expression of *GLN1.1*, *Nii*, *GDH*, *GLU1*, and *GLT* characterized the genetic response of accessions to nitrate starvation, with or without G × N interactions.

**Figure 3:**
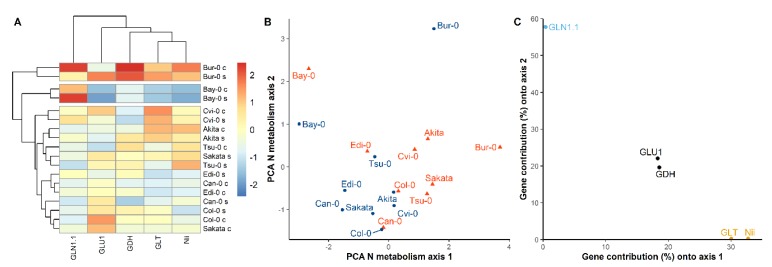
Classification of Arabidopsis accessions following their relative expression of N metabolism-related genes. (**A**) Heatmap of gene expression measured in nine accessions grown in the control or N starvation conditions. Red and blue represented relative high or low gene expression among genotypes. Genotypes were grouped based on the similarity of their expression. For each condition, the matrix of gene expression was centered and scaled over all accessions. (**B**) PCA analysis of expression of N metabolism-related genes. Blue dots and red triangles show the plants grown in the control and N starvation conditions, respectively. (**C**) Contribution of genes to the two first PCA components. The genes contributing above 4.5% to the first, second, or both components are respectively in orange, black, or blue.

To characterize the genetic plasticity among accessions, we classified the means of gene expression of each accession centered and scaled under the two conditions. The heatmap revealed the strong genetic effect on the N metabolism gene expressions since the clustering tree grouped most of the accessions by genotype and not by nutritive condition (Figure 3A). The heatmap isolated clearly the two extreme accessions *Bur-0* and *Bay-0* that showed the highest and lowest gene expressions respectively for all the genes, except *GLN1.1*, which had higher levels of transcripts in *Bur-0* and *Bay-0* than in any of the others accessions. We then run a PCA with the relative gene expression of *GLN1.1*, *Nii*, *GDH*, *GLU1*, and GLT (Figure 3B,C). The first PCA axis (Axis 1; Figure 3B) explained 48.6% of the global variation. The *GLU1*, *GDH*, *GLT* and *Nii* contributed the most to this component (Figure 3C). Although Axis1 did not separate totally the samples corresponding to the two conditions into two distinct groups, it clearly opposed the two conditions for each accession (Figure 3B). Projected distance onto this first component from an accession grown in the control condition to the same accession grown in the N starvation condition was always positive, corresponding to a global increase of expression of N metabolism-related genes (Figure 3B). The second axis (Axis 2, Figure 3B) explained 29.5% and it separated the samples following their genetic origin. Only *GLN1.1*, *GDH* and *GLU1* contributed to this second component (Figure 3C), confirming the importance of these three genes in the strong G × N identified in ANOVA analysis.

### 3.2. The Relative Expressions of 18 Autophagy Genes Were Higher under Nitrate Starvation Than in the Control Condition

The relative expression of 24 autophagy-related (ATG) genes was monitored (Supplementary Table S3). Interestingly, most of these *ATG* genes showed a higher expression level under N starvation condition than in control condition (Table 2). The transcript levels of five of them (*ATG1b*, *ATG7*, *ATG8b*, *ATG8c*, *ATG8d* and *ATG8e*) were not significantly affected by the 24 h of nitrate starvation. The others responded to nitrate starvation by enhanced expression, ranging from +19% for *ATG4a* to more than +60% for *ATG8h* and *ATG9*.

### 3.3. The Collection of Natural Accessions Revealed Genetic Variation of the Relative ATG Gene Expressions

**Figure 4:**
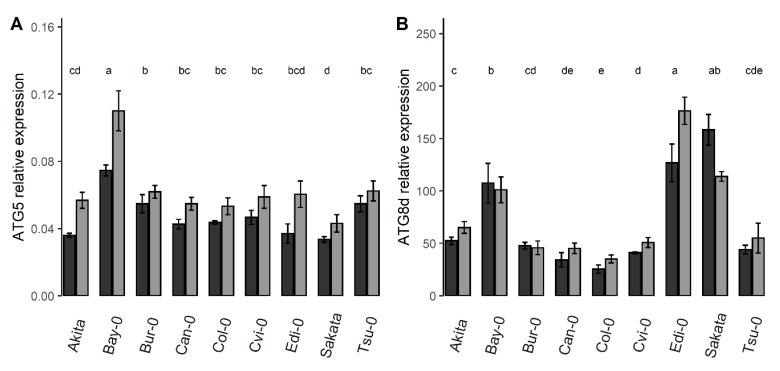
Genetic effect on the *ATG* transcript levels among accessions in the control and starvation conditions. Bars are the relative expressions of *ATG5* (**A**) and *ATG8d* (**B**) in nine Arabidopsis accessions grown in the control and N starvation conditions (means ± SE). Letters indicate significant distinct groups of accession (*p* < 0.05, *n* = 8). Dark and light greys show the plant in the control and N starvation conditions, respectively.

The ANOVA analysis had shown that most of the *ATG* genes showed significant genotype-related variations (Figure 1B). However, unlike the N metabolism-related genes presented above, for which the genotype factor explained the largest part of the variation of their mRNA accumulation, the weights of genetic factor onto the transcript levels of the various *ATG* genes were quite variable. It explained less than 15% of the observed variation for *ATG4a*, *ATG4b*, *ATG8i*, *ATG9*, and *ATG10*, but more than 65% of the observed variation for *ATG5*, *ATG8c*, *ATG8d*, and *ATG12a*. For instance, the relative expression of *ATG5* was much higher in *Bay-0* than in all the other accessions and lower in *Sakata* (Figure 4A). This was different for *ATG8d* whose relative expression was sharply highest in *Bay-0*, *Edi-0* and *Sakata* than in the other accessions (Figure 4B). Indeed, the accessions showing the highest relative gene expressions were different depending on each *ATG* gene.

**Figure 5:**
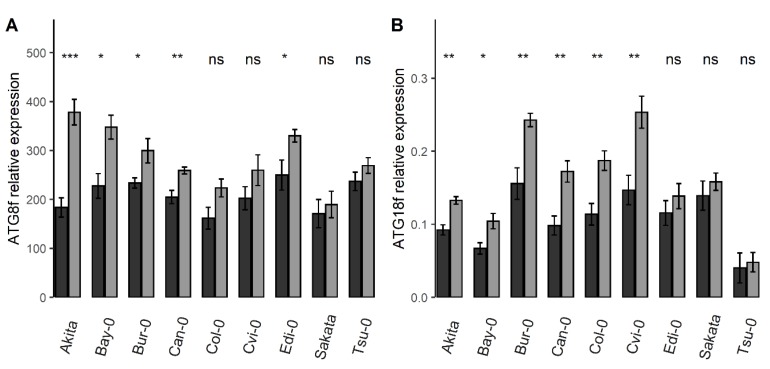
Genetic by Nutrition (G × N) interaction effect on the *ATG* transcript levels. Bars are the relative expressions of *ATG8f* (**A**) and *ATG18f* (**B**) in nine Arabidopsis accessions grown in the control and N starvation conditions (means ± SE). Significant differences between groups are indicated by *asterisks* (* *p* < 0.05, ** *p* < 0.01, *** *p* < 0.01, *n* = 8). Dark and light greys show the plant in the control and N starvation conditions, respectively.

In Figure 1B, the ANOVA had shown significant G × N interactions for 13 *ATG* genes (*ATG1a*, *ATG1b*, *ATG5*, *ATG7*, *ATG8a*, *ATG8b*, *ATG8c*, *ATG8d*, *ATG8f*, *ATG8g*, *ATG8i*, *ATG12a* and *ATG18f*), thus revealing that the level of response of the *ATG* genes to nitrate starvation varied among accessions. The Figure 5 illustrates this G × N interaction for *ATG8f* and *ATG18f*. We measured an overall increase of the relative expression of *ATG8f* and *ATG18f* in the N starvation condition compared to the control condition (Table 2). The increase of *ATG8f* expression was (i) significant and sharp in *Akita* and *Bay-0* accessions (106% and 53%, respectively), (ii) significant but lower (less than 40%) in other accessions and (iii) suggested but not significant in *Col-0*, *Cvi-0*, *Sakata*, and *Tsu-0*. In similar manner, the increase of *ATG18f* expression was significant in almost all accessions; except in the three accessions *Edi-0*, *Sakata,* and *Tsu-0*. The rise of *ATG18f* expression under N starvation was especially important in *Bur-0* and *Cvi-0*. Therefore, although results showed a general trend of increase of the relative expression level of *ATG* genes under N starvation, data mainly revealed that genetic variations were mostly independent of the growth conditions.

**Figure 6:**
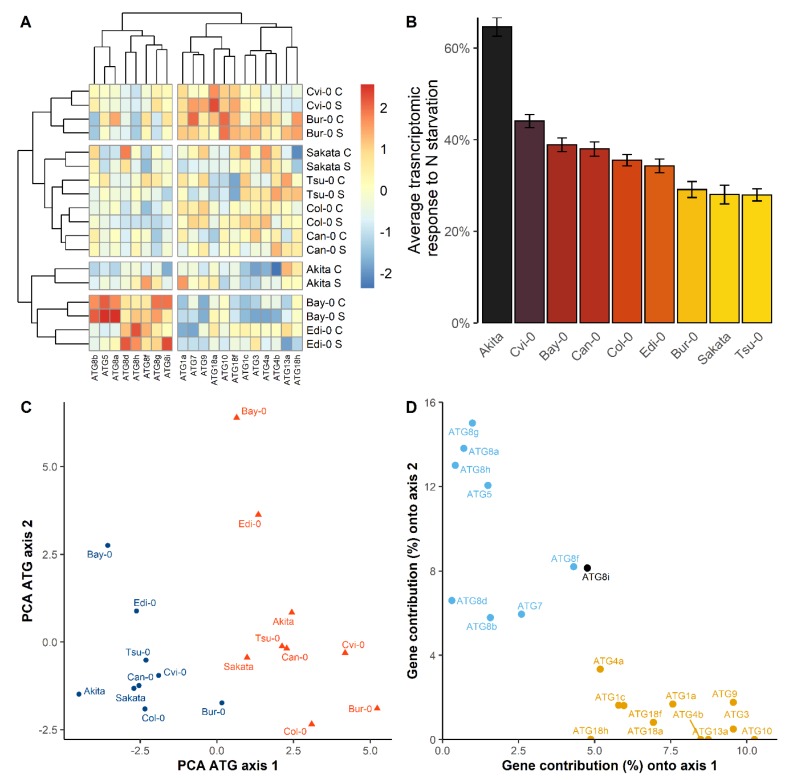
Classification of Arabidopsis accessions following their relative expressions of ATG genes. (**A**) Heatmap of ATG gene expressions measured in nine accessions grown in the control (c) or N starvation (s) conditions. Red and blue represented relative high or low gene expressions among genotypes. Genotypes were grouped based on the similarity of their expressions. For each condition, the matrix of gene expression was centered and scaled over all accessions. (**B**) Average transcriptomic response to N starvation of the accessions (mean ± SE) on the 22 genes. The bars are the average differences for each genotype between the expression values in the control and N starvation condition in percentage of value expression in the control condition. (**C**) PCA analysis of the expression of ATG genes. Blue dots and red triangles show the plants grown in the control and N starvation conditions, respectively. (**D**) Contribution of *ATG* genes to the two first PCA components. The genes contributing above 4.5% to the first, second, or both components are respectively in orange, black, or blue.

Considering that the global response of the ATG genes to the N starvation was genotype-dependent, we aimed at characterizing the specificity of each accession. We then clustered the matrix of the gene expression, scaled and centered by gene and nutritive conditions, selecting the 22 genes that responded to the growth conditions, with or without G × N. The heatmap that resulted from these data then showed that the clustering of the samples separated genotypes and not N nutrition (Figure 6A). Clustering then showed that the genetic diversity observed in the control condition remains similar in the N starvation condition. Some accessions were clearly distinguishable from the others. The *Bay-0* and *Edi-0* exhibited the higher expression values for several members of the *ATG8* family and for ATG5 gene, whereas values for the other genes where globally the lowest as compared to the other accessions. *Akita* showed globally low *ATG* expression levels in the two conditions, while *Cvi-0* and *Bur-0* were mostly characterized by the high expressions of all the *ATG* genes in the two N conditions.

The global response of the nine accessions to nitrate starvation was then evaluated by computing for each accession the average differences of the expression levels of all the *ATG* genes between the control and N starvation conditions. This global difference of expression level was then normalized as percentage of the value expression in the control condition (Figure 6B). The global transcriptomic response to nitrate starvation was the highest in the *Akita*, *Cvi-0*, and *Bay-0* and the lowest in *Sakata*, *Bur-0*, and *Tsu-0*. The highest average transcriptomic response rose to +66.7% in *Akita* accession. Interestingly, phenotype of leaf yellowing was observed solely on the *Akita* plants grown under the nitrate starvation condition (data not shown). We confirmed the early senescence of *Akita* by monitoring the relative expression of the *SAG12* (Senescence Associated Gene 12) gene (Supplemental Figure S1).

PCA was run on the matrix of the expressions of the 22 *ATG* genes (Figure 6C). The first component explained 39.2% of the observed variation and separated well the plants growing under the two nutritive conditions (Axis 1). The second component (Axis 2) explained 23.9% and opposed mainly *Bay-0* and *Edi-0* from the other accessions. Twelve genes contributed to the first component. All the genes showing a strong effect of G × N interaction in ANOVA contributed to the second component (Figure 6D). On the first PCA axis, in the two nutritive conditions, *Bur-0* and *Cvi-0* accessions showed the highest values while *Akita*, *Bay-0*, and *Sakata*, showed the lowest. Interestingly, the projected position of accession *Bur-0* in the control condition on the axis 1 is close to those of *Sakata* and *Bay-0* in the N starvation condition. This result suggested that the overall expression of *ATG* genes in *Bur-0* in the control condition is as high as that in *Sakata* and *Bay-0* in the starvation condition.

### 3.4. The Diversity of N Metabolism Gene Expressions and ATG Gene Expressions Are Correlated in the Two Nutritive Conditions

**Figure 7:**
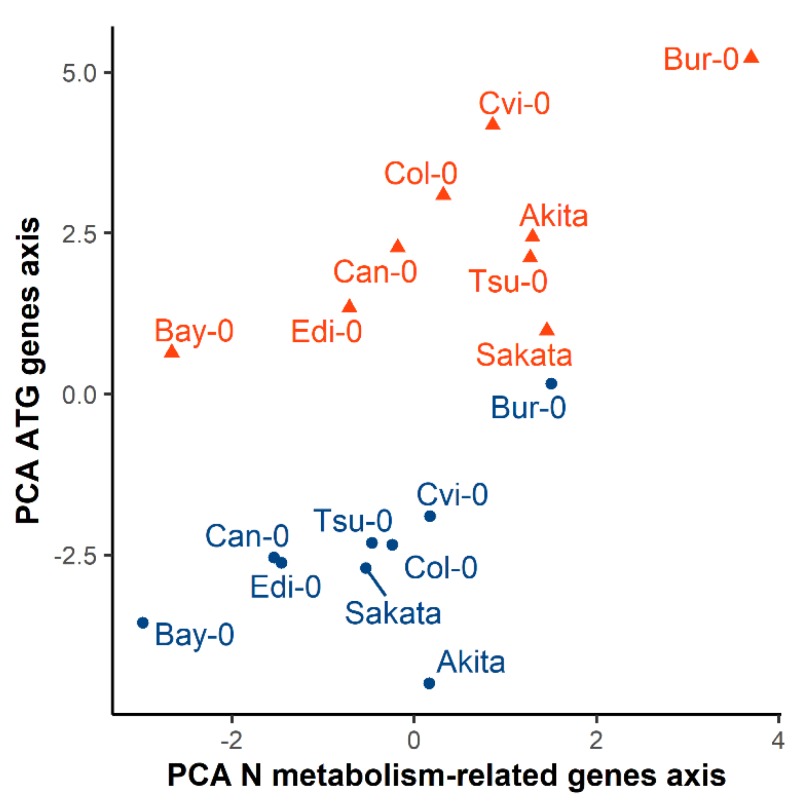
Comparison of global genes expressions among the nine Arabidopsis accessions following the groups of N metabolism related genes and ATG genes. The accessions are distributed in the two conditions following their coordinates onto the PCA N metabolism-related genes axis 1 and the PCA ATG genes axis 1. Blue dots and red triangles show the plants grown respectively in the control and N starvation conditions.

Finally, we investigated whether the strong genetic diversity of *ATG* gene expression is associated with the plant response to N starvation. To address this issue, we compared the genetic diversity of the two biological processes: autophagy and N metabolism. We assumed that the first PCA axes in the two PCA made with N metabolism-related and *ATG* gene expressions presented in Figure 3C and Figure 6C, were linear combinations underling the genetic diversity of the response of the genes to N starvation. Therefore, we recorded the coordinates obtained for each accession on the axis 1 of each PCA and plotted the coordinate values of *ATG* genes against the coordinates of the N metabolism-related genes (Figure 7). The whole set of data obtained on control and starvation condition revealed positive correlations between *ATG* gene coordinates and N metabolism gene coordinates (*r* = 0.62). Similar positive correlation was obtained in each nutritive condition separately (*r* = 0.56 and 0.71, respectively in the control and starvation conditions). The accessions showing the lowest expression levels of *ATG* genes, such as *Bay-0*, *Can-0,* and *Edi*-0, showed the lowest expression levels of N metabolism related genes, irrespective of the nutritive condition. The accessions showing the highest levels of N metabolism transcripts, such as *Bur-0*, had the highest *ATG* gene expression in the two conditions. We concluded that the plasticity in *ATG* gene expression within accessions was associated with the diversity of expression of genes involved in the ammonium assimilation.

## 4. Discussion

### 4.1. Coordinated Regulation of ATG Genes in Response to N Stress Condition

The macro-autophagy process is a vesicular mechanism involving 38 genes in Arabidopsis [29]. We recorded the relative expression level of 24 *ATG* genes in the rosette of plants grown under constant nitrate-rich condition (control) or submitted to N starvation for 24 h. We investigated nine Arabidopsis accessions to estimate the transcriptional plasticity of autophagy. In both ANOVA and multivariate analysis, the nutrition and genotype effects on the variations of gene expressions were significant. In ANOVA, the nutrition and genotype explained on average 14% and 37% of the observed variations respectively (Figure 1B). In PCA, the first principal component opposed the genotypes between the 2 nutritive conditions and explained 41% of the observed variation (Figure 6C). The expression level of the ATG genes was globally higher in the plants submitted to N starvation (Figure 3A–C). On average across all genotypes, no *ATG* transcripts abundance was lower in the starvation condition. The results were consistent with the studies showing the induction of autophagy under C and N limiting conditions [30,36,37]. Besides Arabidopsis, autophagy was shown to be induced in response to nitrogen deficiency in barley [43,44], foxtail millet [45], maize [46] and apple [47,48,49,50]. The global induction of several *ATG* genes by N starvation strongly suggests the existence of a coordinated regulation of the expression of ATG genes in response to nutritive starvation. The C/N balance is reported to regulate post-translationally the autophagy in response to nutriment and sugar deficiency in plants [51]. In low nutriment availability, the SnRK1 complex is activated whereas the TOR kinase complex is inhibited allowing the positive post-translational regulation of the autophagy activity [52]. Recent reports showed the phosphorylation of ATG proteins by TOR complex in a large-scale phosphoproteomics screen [53]. Recently, the transcription factor TGA9 has been reported as a master regulator of *ATG8b* and *ATG8e* gene expressions in response to C deficiency and to osmotic stress [38]. However, the molecular regulators involved in the up-regulation of autophagy gene expression by N limitation remains unknown. It could be an indirect effect on the C/N balance via the TOR pathway or an direct effect of the N pathway via some key actors of the nitrate response, such as NLP, TGA1 or TGA4 transcription factors [38,54].

Among the proteins involved in the core-machinery of autophagy, some ATG proteins are coded by a single gene whereas others are coded by small gene families. We have observed a great diversity of response levels in the collection of the 20 *ATG* genes differentially expressed during nitrate deficiency. The list of genes responding the most strongly to the N starvation included some genes that are unique in the autophagy process (like *ATG3*, *ATG5*, *ATG9*, and *ATG10*) and some specific members belonging to *ATG* gene families (Table 2). On the three members of the *ATG1* family, only *ATG1a* and *ATG1c* were differentially expressed between the two conditions. The two members of *ATG4* family also differed in their degree of response since *ATG4b* was more enhanced than *ATG4a*. Among the three *ATG18* members investigated, *ATG18f* showed the highest response. Interestingly, five of the nine members of the *ATG8* family (*ATG8a*, *ATG8f*, *ATG8g*, *ATG8h* and *ATG8i*) highly responded to nitrate starvation, while the expression of other members was little or not modified by N stress. This could be explained by the fact that some ATG8 isoforms are involved in non-canonical autophagy processes and then not regulated as others [55,56,57]. The high differential expression of genes, which are present in single copy in the Arabidopsis genome, support the hypothesis of a general transcriptional activation of autophagy in the N stress condition. In contrast, the absence of differential expression of the unique *ATG7* gene could be surprising, but it may be explained by a strong regulation of its mRNA stability. In human, the binding of *mir190A* to the 3′-UTR of *ATG7* mRNA regulates its stability [58]. Conservation of similar mechanism to control of *ATG7* expression remains to be discovered in plants.

The activation of particular members of gene families, such as *ATG1*, *ATG4* and *ATG8* families, underlines their importance in the response to N starvation. The abundance transcripts of *ATG8* members is notable since the ATG8 proteins are well known to be at the basis of the selectivity of the autophagy by interacting with specific cargo materials [59]. The *ATG8* genes enhanced by C starvation are almost the same members as we found in the present study to respond to N starvation, with some exceptions. In our condition, the N limiting condition did not affect *ATG8b* expression but enhanced *ATG8f* expression (Table 2), while previous studies report an enhancement of *ATG8b* expression, without any effect on *ATG8f* expression, in both the N limited and fixed-carbon starvation conditions [36,38]. Difference in activation of ATG8 transcripts may due to difference in plant growth conditions and the duration of the stress. Moreover, in most studies about autophagy in Arabidopsis, the accession *Col-0* was chosen as reference, while in our study the effect of nutrition was estimated on our whole collection. Variation between of the global trend observed in a collection of genotypes and the specificity of a single accession may generate the differences in the observation. Nevertheless, from the consistent finding of the response of *ATG8* genes to the C and N limiting conditions, we hypothesized that *ATG8a*, *ATG8g*, *ATG8h* and *ATG8i* are involved in the nutritional response to the C/N balance while *ATG8b* accumulation is specific of the C limiting conditions and *ATG8f* is specific of the N limiting conditions. We noticed that *ATG8h* was the most highly induced by N starvation in here and in previous studies [36,38]. Interestingly, this gene has lower similarity to other members of *ATG8* family. It possesses an exposed Gly residue essential for lipidation, and is thus not supposed to be subjected to cleavage by ATG4 as the other ATG8 members [32]. Moreover, ATG8h is well known to interact with several cargo proteins, such as the ATG8-interaction protein ATI1 in Arabidopsis [55] or plant cell death suppressor ADI3 in tomato [60], suggesting that protein is a key actor of the response to the C/N balance.

### 4.2. Transcriptinal Diversity of ATG Genes among Arabidopsis Accessions

We investigated the genetic diversity of *ATG* genes among nine Arabidopsis accessions. We first noticed a genetic variation of the accumulation of the *ATG* transcripts, even in the control condition. Almost all tested genes showed a genetic variation, independently of the nutrition factor (Figure 1B). In the PCA analysis, the natural diversity is even more obvious (Figure 6C). In the two nutritive conditions, the accessions *Akita*, *Sakata,* and *Bay-0* showed a lower level of *ATG* transcript accumulation than other accessions. By contrast, *Bur-0* and *Cvi-0*, presented globally high *ATG* transcript levels. We concluded that the basal autophagy activity naturally varies depending on the genotype. This plasticity of basal autophagy was enhanced by a natural variation for the overall increase of *ATG* genes during the N deficiency (Figure 6B). The average of transcriptomic increase across all *ATG* genes was more than 40% in accessions *Akita*, *Cvi-0* and *Bay-0* whereas it was less than 30% in accessions *Bur-0* and *Tsu-0*, suggesting that the three first accessions responded more strongly to N starvation than the latter accessions.

We noticed that three accessions *Bay-0, Edi-0* and *Akita* had particular patterns of *ATG* expression (Figure 6A). The accession *Bay-0*, and in a latter extend *Edi-0*, accumulated together more transcripts of *ATG5* and of several *ATG8* members, but they had relatively fewer transcripts of other *ATG* genes compared to the other accessions. The transcripts of almost all *ATG* genes are relatively less in the accession *Akita*. To explain these genetic variations, we hypnotized that the autophagy was less efficient in *Bay-0, Edi-0*, and *Akita* compared to the other accessions. Indeed, it has reported that autophagy mutants (*atg5, atg9, atg18a*, *atg10*) had higher *ATG8* transcript levels than wild type although they are autophagy deficient [36,61,62]. This could be because the absence of N-recycling is sensed as N deficiency in these mutants. The observation of early senescence phenotype (data not shown) and relative accumulation of senescence associated gene *SAG12* in the accessions *Akita* and *Bay-0* (Supplemental Figure S1) support this hypothesis since the rosettes of Arabidopsis *atg* mutants show also early senescence phenotype [23,33].

The demonstration of large natural variation for autophagy, and the description of accessions with high or low autophagy, indicate that it could be possible to modulate the expression level of *ATG* genes (and possibly autophagy activity) avoiding the dramatic effect of *k.o.* mutants or the use of plants with over-expressing genes.

### 4.3. Autophagy Is Part of the Integral Response of the N Metabolism to N Availability

One aim of our study was to determine whether the natural diversity in the ability of genotypes to enhance gene expressions in response to N starvation could provide some advantage/disadvantage to the adaptation of Arabidopsis to nitrogen scarcity. Polymorphisms in *ATG* genes in animal have been observed and associated with disease resistance. For instance, a genome-wide association study in human identified polymorphisms in *ATG* gene, *ATG16L*, associated with differences in disease pathogenesis [63]. In worms, the diversity of *LLG-2*, an homologous gene of Arabidopsis *ATG8* genes, is correlated with the microsporidia colonization inside intestinal cells [64]. In our present study, we did not look for genomic polymorphism of *ATG* genes in our collection of accessions. However, the sequenced genomes of six studied accessions (*Cvi-0*, *Can-0*, *Col-0*, *Edi-0*, *Bur-0*, and *Tsu-0*) are available in the 1001 genome database [65]. Within the sequences, we checked the polymorphism in *ATG* genes in the six accessions but only the accession *Bur-0* showed polymorphisms in the two genes *ATG8a* and *ATG8e*. The lack of massive detected polymorphism in the coding sequences of *ATG* genes suggest that the recorded variability of mRNA accumulation is due to variation of regulators or polymorphisms in the promoter region.

Autophagy is well known to be involved in the regulation of plant growth in response to environmental stress [26]. On one hand, disruption of *ATG* genes results in early leaf senescence and low N remobilization efficiency from the rosette to the seeds in Arabidopsis [33], whereas enhanced autophagy leads to increased seed yield under nitrate limitation and allows a better N remobilization under full nitrate conditions [34]. On the other hand, there is a large natural variation for plant adaptation to N starvation and N limitation [17,18,19], as well as for the N uptake and N remobilization efficiency among Arabidopsis accessions [21,22]. In the present study, we evaluated the plant adaptation to nitrate starvation by monitoring the transcripts accumulation of 11 genes involved in N metabolism. In addition to the strong genetic difference between accessions, we showed that five genes, *GLN1.1*, *Nii*, *GDH*, *GLU1*, and GLT, were good markers to characterize the natural diversity for adaptation to N starvation among accessions (Figure 2B). Recently, the expression of *GLU1* was proposed also to be a biomarker for NUE in a collection of 101 Arabidopsis accessions, following the pattern lower expression in the shoots of lines with high NUE [66]. Compared to *Col-0*, *Bur-0* was described as an accession well adapted to N limitation [17,20,21]. At a same nitrogen nutrition index, defined as the ratio of the N amounts of plants growing in deficient and sufficient conditions, *Bur-0* was an efficient accession for shoot biomass production whereas *Col-0* was an inefficient accession [18]. Since *GLN1.1*, *GDH* and *GLU1* transcripts were highly accumulated in *Bur-0* accession in the N starvation condition (Figure 2A), our results did not support the hypothesis of Meyer et al. [66].

We compared the results obtained in this study with published transcriptomic data on the response of genes to nitrate starvation in supplemental Table S4. In this table, we added our results obtained first on the average of all the accessions (from Table 1; Table 2), and second in the specific *Col-0* accession (from Supplemental Tables S2 and S3), since *Col-0* is commonly used as a reference in the literature. From Table S4, we conclude that:

(i) the results of our study are consistent with the other experiments,
(ii) the average on the nine accessions allowed us to obtain more congruent results than with a single accession, supporting then the relevance of our approach based on the natural diversity,
(iii) our study provides new results, in particular for *ATG* genes, that remain poorly documented in the published transcriptomic data cited in Table S4.

The small heterogeneity between transcriptomic studies supported the previous observation reported by Prof Gutiérrez’s team, which was that the response of “biological process” Gene ontology terms to nitrate deficiency is less sensitive to the experimental context than the gene expressions [67]. In the same way as authors proposed compare gene expressions between studies using the “biological processes”, we have synthetized the response of our genes to nitrate starvation differentiating N metabolism and autophagy as the two biological processes. This was done by working on the PCA axis that are linear combinations of gene expressions that reduced the heterogeneity obtained for one particular gene. This strategy provided a score for each accession, in the two conditions, and for the two processes. Considering that the first components of PCA for *ATG* and N metabolism genes summarized the global diversity of accession for autophagy and of N metabolism, we observed a strong positive relationship between the level of accumulation of the mRNA of *ATG* genes and the level of accumulation of mRNA of N enzymes, regardless the nutrition status (Figure 7). Accessions, such as *Bay-0* and *Edi-0*, have naturally low transcript levels of *ATG* and of ammonium assimilation-related gene compared to accessions such as *Bur-0* (Figure 7). Interestingly, the high level of *ATG* transcripts in *Bur-0* might be well associated with its high NUE [17,20]. Indeed, the accession *Bur-0* previously showed an enhanced remobilization compared to other accessions such as *Col-0* and *Edi-0* [22]. *Bur-0* displayed phenotypes that could remind the *ATG8* over-expressing lines analyzed by Chen et al. [34] that displayed higher N remobilization from the rosette to the seeds and higher N% in seeds under normal N conditions. Altogether, results suggest that the basal activity of autophagy could be associated with capacity of genotypes to be adapted to low N availability. Further experimentations now need to be carried out to explain the observed relationships. Several factors may affect the expression of ATG genes and N remobilization genes. The duration of the N starvation, the kinetics of gene expression, the circadian rhythm, aging, and natural senescence are all well-known external and internal regulators of autophagy and central metabolism that could be investigated.

## Figures and Tables

**Table 1:** Averages on all accessions of differential gene expression between the control and starvation conditions.

Gene Name	AGI Code	Control		Starvation		Differential Variation	*p*-Value	Significant Difference
		Mean	Sd	Mean	Sd			
*NRT2.1*	At1g08090	0.011	0.007	0.009	0.005	−19.0%	0.050	ns
*NRT1.1*	At1g12110	0.119	0.038	0.117	0.044	−1.0%	0.861	ns
*NIA2*	At1g37130	1.651	0.789	1.541	0.743	−6.7%	0.398	ns
*Nii*	At2g15620	1.817	0.560	2.055	0.541	13.1%	0.013	*
*GS2*	At5g35630	4.844	1.492	5.162	1.443	6.6%	0.204	ns
*GLN1.1*	At5g37600	0.335	0.318	0.409	0.272	21.9%	0.146	ns
*AMT1.1*	At4g13510	0.156	0.100	0.165	0.101	5.9%	0.591	ns
*AMT1.4*	At4g28700	0.003	0.002	0.004	0.002	19.5%	0.104	ns
*GDH1*	At5g18170	0.066	0.030	0.079	0.022	19.4%	0.005	**
*GLU1*	At5g04140	6.208	1.835	6.972	2.141	12.3%	0.026	*
*GLT*	At5g16150	0.284	0.110	0.372	0.139	31.0%	0.000	***

* *p* < 0.05, ** *p* < 0.01, *** *p* < 0.001, ns = not statistically significant.

**Table 2:** Averages of differential expression of ATG genes between the control and starvation conditions.

Gene Name	AGI Code	Control		Starvation		Differential Variation	*p*-Value	Significant Difference
		Mean	Sd	Mean	Sd			
*ATG1a*	At3g61960	5.727	1.943	7.841	2.455	36.9%	0.000	***
*ATG1b*	At3g53930	0.034	0.008	0.033	0.010	−3.7%	0.395	ns
*ATG1c*	At2g37840	0.157	0.088	0.215	0.083	36.7%	0.000	***
*ATG3*	At5g61500	0.368	0.125	0.509	0.117	38.4%	0.000	***
*ATG4a*	At2g44140	0.053	0.019	0.063	0.016	18.9%	0.002	**
*ATG4b*	At3g59950	0.117	0.046	0.164	0.036	40.0%	0.000	***
*ATG5*	At5g17290	0.047	0.016	0.063	0.019	33.9%	0.000	***
*ATG7*	At5g45900	8.386	2.284	8.830	2.523	5.3%	0.280	ns
*ATG8a*	At4g21980	0.500	0.222	0.702	0.244	40.3%	0.000	***
*ATG8b*	At4g04620	22.159	10.227	22.614	13.182	2.1%	0.820	ns
*ATG8c*	At1g62040	65.795	34.432	73.977	39.545	12.4%	0.196	ns
*ATG8d*	At2g05630	66.023	51.705	71.989	46.136	9.0%	0.474	ns
*ATG8e*	At2g45170	0.060	0.044	0.069	0.040	15.3%	0.199	ns
*ATG8f*	At4g16520	214.773	63.636	280.682	79.545	30.7%	0.000	***
*ATG8g*	At3g60640	24.148	8.381	32.386	11.648	34.1%	0.000	***
*ATG8h*	At3g06420	15.341	7.273	24.545	9.659	60.0%	0.000	***
*ATG8i*	At3g15580	0.733	0.284	1.097	0.386	49.6%	0.000	***
*ATG9*	At2g31260	0.065	0.036	0.106	0.043	62.6%	0.000	***
*ATG10*	At3g07525	0.035	0.013	0.043	0.025	22.6%	0.020	*
*ATG12a*	At1g54210	0.189	0.136	0.297	0.184	56.8%	0.000	***
*ATG13a*	At3g49590	0.247	0.044	0.374	0.053	51.1%	0.000	***
*ATG18a*	At3g62770	0.203	0.099	0.277	0.108	36.4%	0.000	***
*ATG18f*	At5g54730	0.107	0.056	0.162	0.069	52.0%	0.000	***
*ATG18h*	At1g54710	0.188	0.090	0.238	0.093	26.7%	0.002	**

* *p* < 0.05, ** *p* < 0.01, *** *p* < 0.001, ns = not statistically significant.

## Supplementary Materials

The following are available online at https://www.mdpi.com/2073-4409/9/4/1021/s1. Supplemental Table S1: List of primers used. Supplemental Table S2: Results of qPCR of N metabolism related genes among accessions in two conditions. Supplemental Table S3: Results of qPCR of ATG genes among accessions in two conditions. Supplemental Table S4: Comparison with Publicly Available Microarray Data. Supplemental Figure S1: Relative expression of *SAG12* gene in nine accessions grown in the control and N starvation conditions. Blue and red bars are the relative expressions of *SAG12* in nine Arabidopsis accessions grown respectively in the control and N starvation conditions.
