# Supplementary figures and images for: Transcriptional Plasticity of Autophagy-Related Genes Correlates with the Genetic Response to Nitrate Starvation in Arabidopsis Thaliana
Source: Cells. 2020 Apr 20;9(4):1021. doi: 10.3390/cells9041021 (PMC7226452; doi:10.3390/cells9041021)

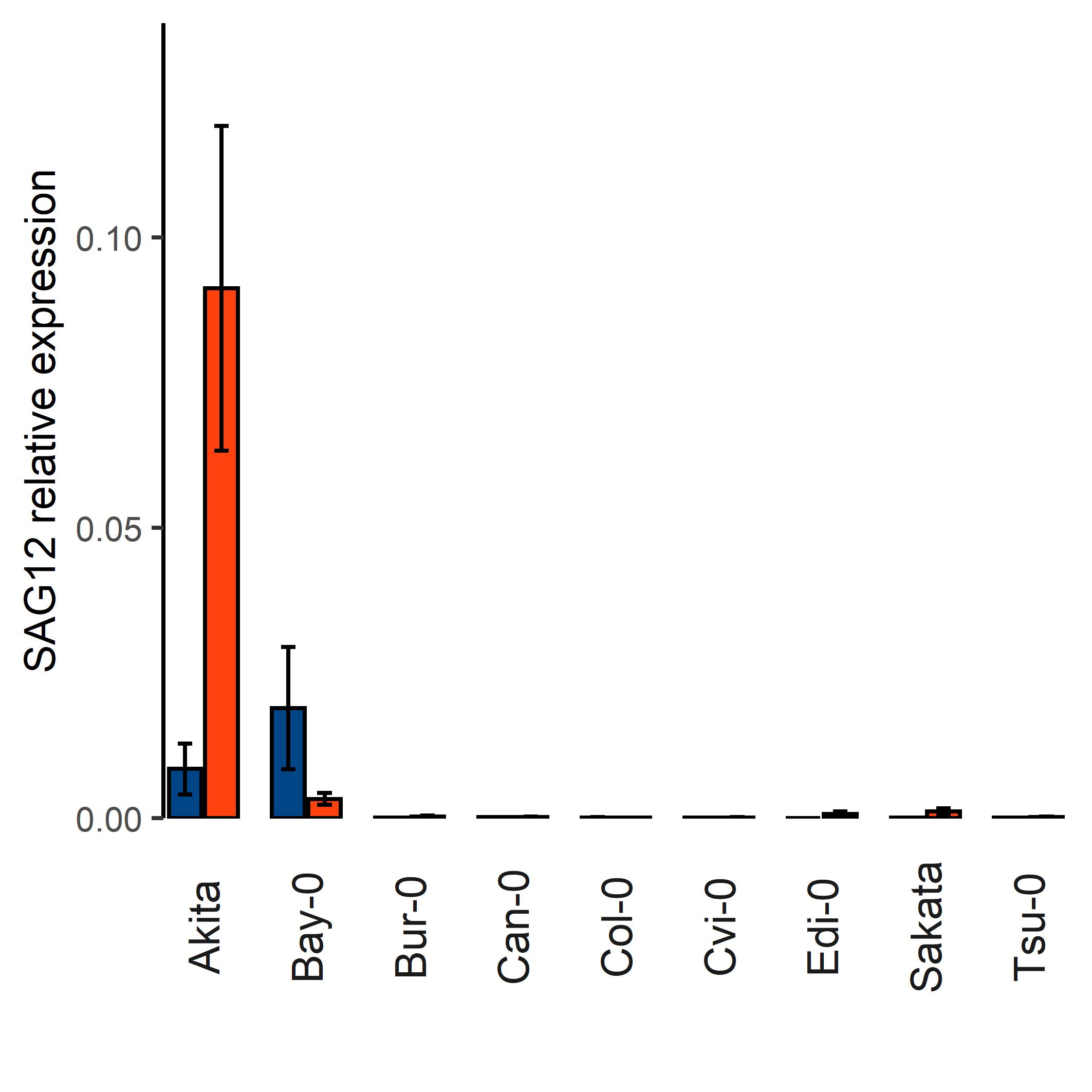

Supplement: Supplementary file 1 [file cells-09-01021-s001.zip › Supplemental Figure S1.tiff]
